# Disentangling genetic risks for development and progression of Alzheimer’s disease

**DOI:** 10.1093/brain/awae237

**Published:** 2024-07-18

**Authors:** Niklas Mattsson-Carlgren

**Affiliations:** Clinical Memory Research Unit, Department of Clinical Sciences Malmö, Lund University, 20502 Malmö, Sweden; Department of Neurology, Skåne University Hospital, Lund University, 22185 Lund, Sweden; Wallenberg Center for Molecular Medicine, Lund University, 22184 Lund, Sweden

## Abstract

This scientific commentary refers to ‘Towards cascading genetic risk in Alzheimer’s disease’ by Altmann *et al.* (https://doi.org/10.1093/brain/awae176).

**This scientific commentary refers to ‘Towards cascading genetic risk in Alzheimer’s disease’ by Altmann *et al.* (https://doi.org/10.1093/brain/awae176).**

Alzheimer’s disease is characterized by the accumulation of amyloid-β (Aβ) and tau pathologies, which show distinct patterns of spatiotemporal progression throughout the brain. Widespread Aβ-pathology occurs early in the disease cascade, and is relatively common in cognitively unimpaired individuals, where it is believed to represent a presymptomatic stage of the disease. In contrast, widespread tau pathology occurs later and correlates with atrophy and cognitive decline. A better understanding of the pathobiological mechanisms that link the development and spread of Aβ and tau may generate opportunities for new therapeutic strategies that can block key steps in the disease cascade. In this issue of *Brain*, Altmann and co-workers^1^ contribute new insights to this field by leveraging genetic risk factors in a longitudinal analysis of individuals who develop biomarker signs of Aβ and tau.

Alzheimer’s disease research has a rich history of genetic studies that have contributed to understanding of disease mechanisms. Rare mutations in three genes intrinsically linked to Aβ-metabolism (*APP*, *PSEN1* and *PSEN2*) have been shown to cause Alzheimer’s disease in an autosomal dominant fashion, by introducing pathological changes in Aβ production. This genetic finding is a substantial component of the amyloid cascade hypothesis, which is the dominant framework for Alzheimer’s disease research. Another genetic risk factor is the ɛ4 allele of the *APOE* gene, which is unique in combining a relatively high frequency with a strong dose-dependent effect on disease risk. Finally, a large number of genetic variants have been identified through genome-wide association studies (GWAS), although most have either very low frequencies or exert only small effects on disease risk. The combined effects of multiple genetic variants can be aggregated in polygenic risk scores (PRS) to facilitate clinical studies. The exact mechanisms by which *APOE* ɛ4 and other genetic variants influence Alzheimer’s disease risk are the subject of extensive research, and are the focus of this study by Altmann and colleagues.^1^

The authors began by analysing a cohort of 312 individuals who were Aβ and tau negative (A−T−) at baseline, and found that over a mean follow-up of 5 years, 65 individuals developed Aβ-positivity (A+T−). A similar analysis was then performed for 290 participants who were Aβ-positive but tau negative (A+T−) at baseline; over a mean follow-up of 4 years, 45 of these individuals progressed to tau positivity (A+T+). The authors then tested whether two genetic factors—*APOE* ɛ4, and a global PRS for Alzheimer’s disease (excluding the *APOE* region)—influenced participants’ risk of transitioning between these biomarker stages.

A key finding was that development of Aβ-positivity (progression from A−T− to A+T−) was associated with *APOE* ɛ4 but not with the PRS. In contrast, development of tau-positivity (progression from A+T− to A+T+) was primarily associated with the PRS, and only weakly associated with *APOE* ɛ4 (Fig. 1). The results were largely consistent when using PRS derived using other Alzheimer’s disease GWAS, and when using different methods to define Aβ and tau status (either CSF biomarkers or PET). Taken together, the results strongly link *APOE* ɛ4 to the initial development of the disease (Aβ pathology), and other genetic variants to downstream disease manifestations (tau pathology). The findings thereby help to disentangle the biological mechanisms that drive different aspects of Alzheimer’s disease.

**Figure 1:**
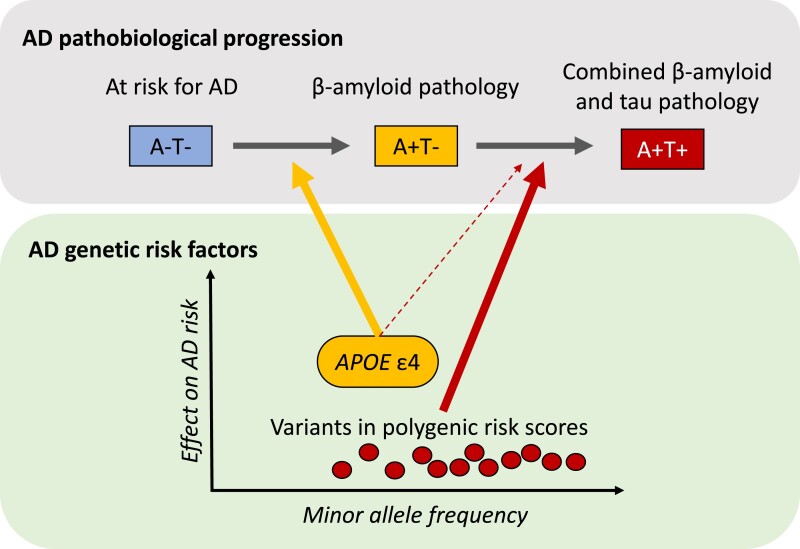
**Linking Alzheimer’s disease genetic risk factors to pathobiological disease progression**. Altmann and co-workers tested the effects of different genetic risk factors on transitions from A−T− to A+T−, and from A+T− to A+T+. They found that *APOE* ɛ4 was mainly associated with transition from A−T− to A+T− (the initial development of Alzheimer’s disease), while an Alzheimer’s disease polygenic risk score was only associated with the transition from A+T− to A+T+. A = amyloid; AD = Alzheimer’s disease; T = tau.

Although the exact role of *APOE* ɛ4 has been debated, the observation by Altmann and colleagues^1^ that *APOE* ɛ4 is the main genetic driver for development of Aβ-pathology in late-onset Alzheimer’s disease is consistent with findings from several previous studies. For example, a study on two large cohorts found that a PRS that included *APOE* ɛ4 predicted much more of the variance in Aβ-burden (∼8%–24%) than a PRS that excluded the *APOE* region (∼2%–4%).^2^ There is also support for the notion that *APOE* ɛ4 has little effect on downstream aspects of Alzheimer’s disease once Aβ-burden is accounted for. A large study focusing on presymptomatic Alzheimer’s disease, for example, found no effect of *APOE* ɛ4 on subtle cognitive decline, once predictive models were adjusted for Aβ-load.^3^ In a study on tau PET, effects of *APOE* ɛ4 on tau were found to be marginal or non-existent once the models were adjusted for Aβ-burden.^4^

The finding that non-*APOE* variants influence later stages of the disease cascade is also consistent with previous literature. For example, several genetic variants have been found to moderate the associations between Aβ-PET and cognition, potentially by altering the brain’s response to Aβ pathology.^5^ Cases of individuals with mutations for autosomal dominant Alzheimer’s disease have shown that rare genetic variants can protect against development of tau pathology, even on strongly dominant genetic backgrounds. Note that these protective variants include the *APOE3* Christchurch mutation, raising the possibility that *APOE* may still be involved, under some circumstances, in the transition from Aβ-pathology to combined Aβ and tau pathology.^6^ Previous analyses of Alzheimer’s disease PRSs found associations with longitudinal cognitive decline that were only partly mediated by Aβ burden, adding support to the notion that PRSs capture effects that extend beyond Aβ pathology.^7^ Several previous studies have also tested associations between PRSs and tau, and found associations both with CSF tau measures,^8^ and with cortical tau PET.^9^

Taken together with the existing literature, the findings of Altmann and colleagues^1^ are logical and compelling. Important novel aspects of the study include its longitudinal design and the integration of genetic analyses into the conceptual framework of Alzheimer’s disease staging using Aβ and tau biomarkers. This allows a new genetic perspective on the temporal evolution of Alzheimer’s disease.

One limitation of the study is the use of a single PRS trained on all variants associated with Alzheimer’s disease diagnosis at a high level of significance. Future studies could usefully fine-tune PRSs for specific genetic components that are particularly linked to tau aggregation. For example, the authors noted that their PRS included variants that have been linked individually to tau aggregation, including a *BIN1* variant, which interacts with Aβ load to accelerate tau accumulation.^10^ Studies that integrate regional gene expression data could add further support to the role of specific genes in the Alzheimer’s disease cascade, for example by testing spatial correlations with regions prone to Aβ or tau aggregation.

Another limitation of the current study is its reliance on a single cohort: the Alzheimer’s Disease Neuroimaging Initiative. Future studies should aim both to verify the reproducibility of the findings, and to extend the analyses for an even more granular understanding of the biological mechanisms linking the key pathological elements of Alzheimer’s disease. To unambiguously clarify the roles of different genetic components during disease progression, such studies could follow individuals during their entire progression from A−T− to A+T+. Although these studies will by necessity require very long follow-up, they will importantly be able to account for the duration of Aβ-positivity (in the current study it is possible that *APOE* ɛ4 positive individuals had a longer duration of Aβ-positivity, contributing to the effects of *APOE* ɛ4 on transition from A+T− to A+T+). To clarify the potential of genetic risk models for personalized medicine and subject-level prognosis, studies are needed that compare genetic risk factors head-to-head with other types of data that can be used for prognostication (e.g. state-of-the-art plasma biomarkers).

In sum, this elegant study by Altmann *et al.*^1^ is an important addition to our understanding of the genetic mechanisms that affect both the onset and progression of Alzheimer’s disease, and paves the way for exciting follow-up projects to further study the genetic architecture of Alzheimer’s disease.
